# Improving Community Health Using an Outcome-Oriented CQI Approach to Community-Engaged Health Professions Education

**DOI:** 10.3389/fpubh.2017.00026

**Published:** 2017-02-27

**Authors:** Amy Clithero, Simone Jacquelyn Ross, Lyn Middleton, Carole Reeve, Andre-Jacques Neusy

**Affiliations:** ^1^Family and Community Medicine, University of New Mexico School of Medicine, Albuquerque, NM, USA; ^2^College of Medicine and Dentistry, James Cook University, Townsville, QLD, Australia; ^3^Training for Health Equity Network, New York City, NY, USA; ^4^School of Health Sciences, University of KwaZulu-Natal, Durban, South Africa; ^5^School of Medicine, Flinders University, Alice Springs, NT, Australia

**Keywords:** social accountability, health professional education, continuous quality improvement, health workforce, accreditation

## Abstract

Health professionals providing health-care services must have the relevant competencies and clinical experiences needed to improve population health outcomes in different contexts. Current models of health profession education often fail to produce a fit-for-purpose workforce ready and willing to provide relevant, quality care to underserved communities. Evidence is emerging that community-engaged and socially accountable health workforce education, i.e., aligned with priority health needs, produces a workforce ready and willing to work in partnership with underserved regions. This model of education fosters greater affiliation between education and service delivery systems and requires institutions to measure graduate outcomes and institutional impact. The Training for Health Equity Network (THEnet), a partnership of socially accountable health workforce education institutions, has developed and tested a Social Accountability Framework for Health Workforce Education (the Framework) and toolkit to improve alignment of health workforce education with outcomes to assess how well education institutions meet the needs of the communities they serve. The Framework links education and service delivery creating a continuous quality improvement feedback loop to ensure that education addresses needs and maximizes impact on the quality of service delivery. The Framework also provides a unifying set of guidelines for health workforce policy and planning, accreditation, education, research, and service delivery. A key element to ensuring consistent high quality service delivery is an appropriately trained and equitably distributed workforce. An effective and comprehensive mechanism for evaluation is the method of CQI which links the design, implementation, accreditation, and evaluation of health workforce education with health service delivery and health outcomes measurement.

## Introduction

The World Health Organization (WHO) estimates that an additional 2.4 million doctors, nurses, and midwives are needed globally but nowhere near enough are being trained, particularly in the areas where they are needed the most; but increased numbers of health professionals is insufficient (1). They need to be equitably distributed, competent to meet the needs of their communities, and be motivated and empowered to deliver quality care that is appropriate and acceptable to the sociocultural needs of the population (2). It is well documented that poverty and social inequity are the most important determinants of ill health worldwide (3, 4). Many, if not all, intractable health problems have as their root cause social determinants, including health inequities between economic and ethnic groups and poor access to health care. Health professionals have a responsibility to address social inequity and its deleterious effects on individual and population health. An appropriately trained and evenly distributed health workforce is essential to reduce the health equity gap within and across borders and to achieve universal health coverage (UHC) and meet the Sustainable Development Goals (SDG 3) (http://www.who.int/topics/sustainable-development-goals/targets/en/).

The Independent Global Commission on Education of Health Professionals for the twenty first century identified that “*glaring gaps and inequities in health persist both within and between countries* … *and* … *professional education has not kept pace with these challenges*” (4). They acknowledge that the problems are systemic and require a new era of health professional education. Specifically, they classified three successive levels of learning for students to build their knowledge, skills, attributes, and values for how to become a health system change agent. These are:

1. Informing: acquiring skills
2. Forming: creating professional identity
3. Transforming: creating leaders who can effectively lead health systems and improve population health

The Global Strategy on Human Resources for Health: Workforce 2030 recommends education strategies include social accountability (SA) approaches to ensure a better distribution of health workers where they are most needed, emphasizing the underserved and most vulnerable populations (2). This is echoed by the recently released report of the High Level Commission on Health and Economic Growth stressing that socially accountable education should be institutionalized emphasizing the role of training institutions in addressing population and health system needs (5).

Further, there is a growing global consensus recognizing the importance of holding health professional schools accountable to society for achieving these goals. The WHO defines SA as “*the obligation to orient education, research, and service activities towards priority health concerns of the local communities, the region and/or nation (schools) one has a mandate to serve. These priorities are jointly defined by government, health service organizations, and the public”* (6). The WHO is not alone in recognizing SA as a critical mandate. The 2010 global consensus on SA document, reflecting the agreement of 130 organizations and individuals from around the world involved in health education, professional regulation, and policy-making, called for schools “*to reorient their education, research, and service priorities*” (7) to improve their response to current and future health-related needs and challenges in society. This requires health professional schools to shift their traditional education model toward a socially accountable approach. Despite this, there are limited practical tools to guide health professional schools to transform their curriculum and measure their impact on health outcomes.

### The Training for Health Equity Network

Driven by both their implicit and explicit social mission to address the needs of their communities, a number of schools of medicine and health sciences in high and low resources countries have embraced this challenge by successfully incorporating SA as the central tenet of their mission. Their success in producing graduates with broader and relevant competencies and distributed equitably in geographically isolated, underserved regions led to the development of the Training for Health Equity Network (THEnet). THEnet was founded in 2008 and is an international collaboration of 12 health professional schools committed to SA mandates to direct their educational, research, and service resources toward the priority health and health system needs of their reference populations (8). The first priority of action for THEnet was to develop and test a Framework for Socially Accountable Health Workforce Education (the Framework) to assist health professional educational schools measure their progress toward SA (9). The Framework was informed by Boelen and Woollard’s three “expressions of social accountability” namely: “*conceptualization (the type of professional needed and the system that will utilize his or her skills), production (the main components of training and learning) and usability (initiatives taken by a school to ensure that its trained professionals are put to their highest and best use)*” (10). Following its publication in 2012, the Framework has been used by a growing number of health professional schools across the world to evaluate their curriculum, or discuss opportunities for education or policy change (11–14).

### SA Health Professional Education and Continuous Quality Improvement (CQI)

**Figure 1:**
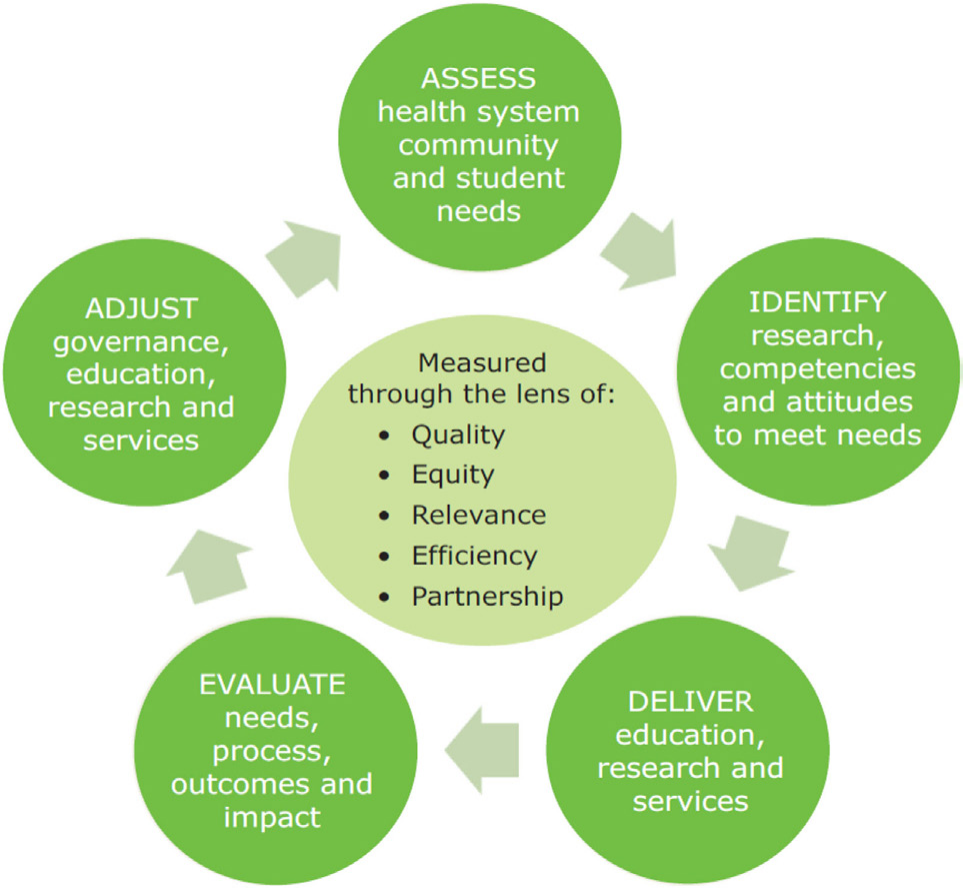
**THEnet iterative continuous quality improvement model**.

Continuous quality improvement as a process method can be used to continually improve the quality of student learners over time which is a step beyond quality assurance which can be viewed as simply producing technically competent graduates. CQI is a set of principles, concepts, and methods adopted originally in the business world and subsequently introduced to other areas including the higher education sector (15). Quality improvement processes build on quality assurance systems in higher education ensuring quality of teaching and learning and providing public accountability for the standards of programs and the use of resources by meeting accreditation standards (16). Traditionally, higher education quality assurance systems such as accreditation bodies focus more on educational processes than on outcomes and impacts of their graduates and research on societal issues and communities they serve (17). THEnet Framework bridges this gap by aligning education processes with the impact of the graduates in the communities. The THEnet iterative CQI model (Figure 1) shows a CQI cycle of health professional education, research, and service within a traditional CQI structure of Plan—Do—Study—Act.

## Methods

The Framework identifies key factors for schools to educate a health workforce to positively influence health outcomes and health systems performance and provides the training and tools to measure and improve the outcomes across institutions and context. The Framework was developed using a logical framework matrix (NORAD 1999), a well-tested project planning and evaluation tool (18). The Framework helps schools evaluate how well they are doing in terms of meeting priority needs and assists to establish educational improvement and areas for research *via* the four sections of the Framework. Each section addresses an element of the CQI cycle by asking practical questions linked to stages of the quality improvement process. The four sections are (1) what needs are we addressing? (2) how do we work? (3) what do we do? and (4) what difference do we make? These four sections of the Framework inform each of the other sections and provide strategies for transformational learning to generate future cycles leading to continuous improvements over time.

### Creating a Health Professional Education Curriculum to Meet the Health Workforce Needs

THEnet’s iterative CQI model (Figure 1) and the four sections of the Framework have been linked to showcase how to create a health professional education curriculum to meet health workforce needs with a continuous evaluation process.

#### Assessment and Identification: Section 1: What Needs Are We Addressing? and Section 2: How Do We Work?

Section 1: the first step is to examine and determine if there is a strong alignment between the school’s community needs and the desired graduate competencies. A socially accountable health professional curriculum considers the geographical region the school serves, communities that have difficulty accessing health services, or have poor health outcomes in the region. Inclusion is a quality CQI step, which means involving key stakeholders including community members in the design of a curriculum for buy-in and quality graduate attributes. Other stakeholders such as learners, educators, community members, health service providers, management, and government also bring different perspectives, knowledge, and necessary information to the process.

Section 2: spend quality time ensuring that the learners, educators, leaders, and key stakeholders are aware of vulnerable populations and underserved communities in the region and can identify their priority health and social needs. This knowledge must then become embedded in the curriculum, and be geared toward transformational learning to produce graduates with the competencies and commitment to address identified priority health, cultural, and social needs of the communities they serve with a focus on the underserved.

#### Deliver: Section 3: What Do We Do?

A socially accountable health professional curriculum considers, what, how, and where do our learners learn, and embeds the values of quality, equity, relevance, and efficiency. It also considers who the educators are and how are they trained, and governance needs such as how resources are managed for program operationalization so they are distributed according to priority needs.

The socially accountable curricula emphasize the principles of primary health care, and integrate basic and clinical sciences with population health and social sciences. The second quality improvement step specific to the curriculum should include who will do the curriculum review, what specifically will be done and when will it be done. Consideration also needs to be given to any tools and training that may be needed. For example, if a change is needed, consideration needs to be given as to whether it is feasible to make the change in terms of cost, time, and resources and if there is buy-in by key decision makers.

Undertaking a comprehensive curriculum review can be daunting so think about the one change that might be worth undertaking. For example, review what your learners learn from your curriculum. Suggested indicators from Section 3 of the Framework include (19):

- Does your education program, including curriculum content, reflect identified priority health, cultural, and social needs of the community?
- Does the learning define the knowledge, attitudes, and skills needed to meet the health needs of the populations and regions served?
- What number or proportion of curriculum weeks are allocated to high priority community health needs?
- Does your curriculum design, delivery, assessment, and evaluation reflect the:
  ▪ desired graduate attributes based on the above needs assessment?
  ▪ principles of primary health care? and
  ▪ integration of basic and clinical sciences with population health and social sciences?

#### Evaluate: Section 4: What Difference Do We Make?

**Figure 2:**
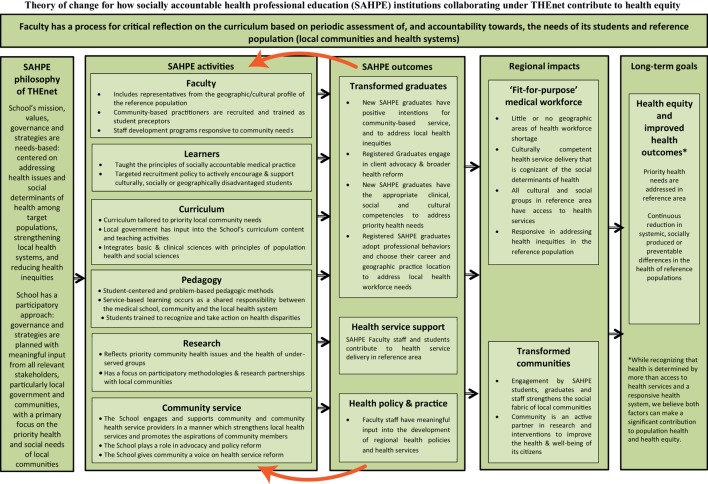
**Theory of change for how socially accountable health professional education (SAHPE) institutions collaborating under THEnet contribute to health equity (28)**.

The next stage is monitoring the curriculum for impact. Before evaluating, consider the processes, strategies, outcomes, and the impact that curriculum reform will have on the systems, communities, and individuals it serves. To help with this process, a program logic model can be developed to identify if the needs will be met (20). As an example, THEnet program logic model (Figure 2) outlines THEnet’s socially accountable health professional education (SAHPE) philosophy, activities, outcomes, regional impacts, and long-term goal of health equity and improved health outcomes. Other evaluation tools can include student satisfaction surveys with learning, graduate competency surveys within the health system (patient and supervisors), and faculty satisfaction with institutional support for undertaking curriculum initiatives toward SA surveys.

Impact factors might include a broader study of improvement in population health derived from local health surveys and statistics, improvement in health workforce numbers and retention across the region, and number of research publications and conference presentations of socially accountable projects by faculty and students, as well as tracking graduate specialty and practice location to determine if they match the priority workforce needs of the community served.

#### Adjust

The final stage of a CQI process is acting upon what is learned and adjusting governance, education, research, and services accordingly and informs the next iteration of the quality improvement cycle. CQI is an ongoing process and each school must continue to examine their underlying assumptions, and be proactive and responsive to changing needs and demands.

## Discussion

THEnet recognizes that SA is a fundamental principle that requires a flexible approach in how it is operationalized. The Framework is a generic tool and can be used in phases and allows for creativity and adaptability to different contexts and different resource availability. Over the past 4 years, THEnet schools realized the benefit of the Framework, not only for curriculum transformation but also as a mechanism for CQI for the production of a fit-for-purpose workforce to improve local health outcomes.

A significant investment is required by health professional education institutions and society to develop health professionals who have both technical expertise and professional values that include a service orientation and ethical commitment to not only their individual patients but the communities in which they practice. SA is a principle that translates into educational strategies resulting in value-based competencies that are best demonstrated by health professionals who act as change agents in partnership with their communities. THEnet iterative CQI model links the educational and service delivery systems. As a practical CQI tool, THEnet Framework on SA has filled an important gap and is currently being used by a growing number of health professional education institutions around the world. We propose integrating the Institute of Medicine’s definition of CQI with health professional education by including education as a strategy for improving health-care services and by describing targeted patient groups as communities: *“Quality improvement consists of systematic and continuous actions that lead to measurable improvement in education, health care services and the health status of targeted patient groups [communities]”* (21).

## Conclusion

A CQI approach is useful for understanding and monitoring socially accountable educational mechanisms that lead to fit-for-purpose graduates and improved quality of care and health outcomes at a population level. SA in health professional education is gaining traction internationally as a mechanism for combatting health inequities and advancing UHC (1).

Including SA indicators in health professional education accreditation standards would acknowledge the importance of holding health professional schools accountable to society for addressing population and health system needs (5). We call for key indicators around an appropriately trained and evenly distributed health workforce to be included in all health professional higher education accreditation processes. This is essential to reduce the health equity gap within and across borders and to achieve UHC and meet the Sustainable Development Goals (SDG 3) (http://www.who.int/topics/sustainable-development-goals/targets/en/).

THEnet’s Framework for Socially Accountable Health Workforce Education links education and service delivery creating a CQI feedback loop to ensure that education addresses needs and maximizes impact on quality service delivery. Evidence is emerging that community-engaged and socially accountable health workforce education produce a workforce ready and willing to work in partnership with underserved regions (22–27).

## Author Contributions

All authors contributed to the writing and editing of the article. AC wrote the first draft and managed subsequent drafts with revisions from all other authors. SR wrote the second draft. CR and LM reviewed subsequent drafts and provided revisions. A-JN did the final review. All authors gave final approval of the publication of this version of the paper.

## Conflict of Interest Statement

The authors declare that the research was conducted in the absence of any commercial or financial relationships that could be construed as a potential conflict of interest.
